# Spread of tau down neural circuits precedes synapse and neuronal loss in the rTgTauEC mouse model of early Alzheimer's disease

**DOI:** 10.1002/syn.21965

**Published:** 2017-03-06

**Authors:** Eleanor K. Pickett, Christopher M. Henstridge, Elizabeth Allison, Rose Pitstick, Amy Pooler, Susanne Wegmann, George Carlson, Bradley T. Hyman, Tara L. Spires‐Jones

**Affiliations:** ^1^ The University of Edinburgh Centre for Cognitive and Neural Systems, Centre for Dementia Prevention and the Euan MacDonald Centre for Motor Neurone Disease Research 1 George Square Edinburgh United Kingdom; ^2^ McLaughlin Research Institute Great Falls Montana; ^3^ Massachusetts General Hospital and Harvard Medical School, MassGeneral Institute for Neurodegenerative Disease Charlestown Massachusetts

**Keywords:** Alzheimer's disease, array tomography, rTgTauEC, tau

## Abstract

Synaptic dysfunction and loss is the strongest pathological correlate of cognitive decline in Alzheimer's disease (AD) with increasing evidence implicating neuropathological tau protein in this process. Despite the knowledge that tau spreads through defined synaptic circuits, it is currently unknown whether synapse loss occurs before the accumulation of tau or as a consequence. To address this, we have used array tomography to examine an rTgTauEC mouse model expressing a P301L human tau transgene and a transgene labeling cytoplasm red (tdTomato) and presynaptic terminals green (Synaptophysin‐EGFP). All transgenes are restricted primarily to the entorhinal cortex using the neuropsin promotor to drive tTA expression. It has previously been shown that rTgTauEC mice exhibit neuronal loss in the entorhinal cortex and synapse density loss in the middle molecular layer (MML) of the dentate gyrus at 24 months of age. Here, we observed the density of tau‐expressing and total presynapses, and the spread of tau into the postsynapse in the MML of 3–6, 9, and 18 month old red–green‐rTgTauEC mice. We observe no loss of synapse density in the MML up to 18 months even in axons expressing tau. Despite the maintenance of synapse density, we see spread of human tau from presynaptic terminals to postsynaptic compartments in the MML at very early ages, indicating that the spread of tau through neural circuits is not due to the degeneration of axon terminals and is an early feature of the disease process.

## 1 Introduction

The observation that neurons of the entorhinal cortex (EC) are affected very early by neurofibrillary tangle pathology in Alzheimer's disease (AD) has been recognized for >30 years (Braak & Braak, 1991; Hyman, Van Hoesen, Damasio, & Barnes, 1984). These layer II neurons link the cerebral cortex with the hippocampus via the perforant pathway (Hyman, Kromer, & Van Hoesen, 1987), a critical projection for memory function. Disruption of this neural circuit through selective loss of neurons, synapses, and the accumulation of tau lesions, is thought to contribute to the early memory impairments observed in AD (Hyman, Van Hoesen, & Damasio, 1990). As the disease progresses, tau pathology propagates from the EC in a well‐characterized anatomical pattern extending to limbic and association cortices (Braak & Braak, 1991). The mechanism of this spreading has yet to be determined, however, mounting evidence suggests that tau spreads trans‐synaptically and that synaptic activity increases the spread of tau through synapses (de Calignon et al., 2012; Harris et al., 2012; Liu et al., 2012; Pooler, Phillips, Lau, Noble, & Hanger, 2013; Walsh & Selkoe, 2016; Wu et al., 2016). One potential route of tau moving from presynapses to postsynapses is the degradation of presynaptic terminals due to the presence of toxic tau. This would release pathological tau from the degenerating presynaptic terminal which could then be taken up by the postsynapse; a possibility supported by many studies showing that cells in culture can take up extracellular tau (Frost, Jacks, & Diamond, 2009; Guo & Lee, 2011; Kfoury, Holmes, Jiang, Holtzman, & Diamond, 2012; Lewis & Dickson, 2016).

In the present study we directly address the question of whether degeneration of tau expressing presynaptic terminals is necessary for the spread of tau to postsynaptic compartments in a defined neural circuit. To do this, we examine the red–green‐rTgTauEC transgenic mouse model, which reversibly express human mutant P301L tau under the control of the neuropsin promoter restricting expression primarily to the medial entorhinal cortex (de Calignon et al., 2012; Polydoro et al., 2013; Pooler, Polydoro, et al., 2013). These neurons overexpressing mutant tau also express Myc‐tagged tdTomato and full‐length synaptophysin/mut4EGFP fusion protein (EC‐tdTomato/Syp‐GFP) (Li et al., 2010; Miyamichi et al., 2011). tdTomato expression is cytoplasmic, while GFP expression is directed to the presynapse. This mouse line allows visualization of the synaptic terminals of human tau expressing neurons. Here, we utilized array tomography to determine whether the density of tau and GFP‐expressing presynapses is altered prior to global synapse loss. Employing this high resolution imaging technique, we further characterized the spread of human tau protein within this model and demonstrate that the propagation of tau through anatomically connected brain regions is not due to the degeneration of presynaptic terminals and is an early feature of the disease process.

## 2 Material and methods

### 2.1 Animals

Three to six (*n* = 4), 9 (*n* = 4), and 18 (*n* = 4) month old rTgTauEC + EC‐tdTomato/Syp‐GFP and 3–6 (*n* = 3), 9 (*n* = 3), and 18 (*n* = 6) month old EC‐tdTomato/Syp‐GFP littermate control mice of both sexes were used for array tomography experiments. rTgTauEC + EC‐tdTomato/Syp‐GFP mice were generated as described previously (Pooler, Polydoro, et al., 2013) by crossing FVB‐Tg(tetO‐Tau_P301L_)4510 (SantaCruz et al., 2005) mice with the Tg(tetO‐tdTomato‐Syp/mut4EGFP)1.1Luo/J line obtained from Jackson Laboratory . Progeny expressing both Tg(tetO‐Tau_P301L_)4510 and Tg(tetO‐tdTomato‐Syp/mut4EGFP) transgenes were crossed with a line expressing the tetracycline sensitive transcriptional activator controlled by the *Klk8* neuropsin promotor (EC‐tTA) to generate mice expressing both rTgTauEC + EC‐tdTomato/Syp‐GFP. Littermates expressing the EC‐tTA and Tg(tetO‐tdTomato‐Syp/mut4EGFP) to result in EC‐tdTomato/Syp‐GFP without tau expression were used as controls. PCR screening was used to genotype animals using primer pairs 5′‐ACCTGGACATGCTGTGATAA‐3′ and 5′‐TGCTCCCATTCATCAGTTCC‐3′ for the EC‐tTA transgene, 5′‐TGAACCAGGATGGCTGAGCC‐3′ and 5′‐TTGTCATCGCTTCCAGTCCCCG‐ 3′ for Tg(tetO‐Tau_P301L_)4510 tau transgene, and 5′ CTT CAA GTC CGC CAT GCC CGA 3′ and 5′ TCC AGC AGG ACC ATG TGA TCG C 3′ for the EC‐tdTomato/Syp‐GFP transgene.

All animal experiments were performed in accordance with institutional and national ethics guidelines and approved by the Harvard Medical School Institutional Animal Care and Use Committee and the U.K. Home Office.

### 2.2 Array tomography

Fresh brain tissue samples were collected from rTgTauEC + EC‐tdTomato/Syp‐GFP and EC‐tdTomato/Syp‐GFP control transgenic mice as outlined previously (Kay et al., 2013; Koffie et al., 2009). Briefly, small tissue blocks containing the dentate gyrus and entorhinal cortex were fixed in 4% paraformaldehyde and 2.5% sucrose in 20 mM phosphate buffered saline pH7.4 (PBS) for 3 hr. Samples were then dehydrated through ascending cold graded ethanol and embedded into LR White resin (EMS) which was allowed to polymerize overnight at 53°C. Resin embedded tissue blocks were cut into array ribbons of 70 nm thick sections using an ultracut microtome (Leica) equipped with a Jumbo Histo Diamond Knife (Diatome, Hatfield, PA) and collected onto gelatin coated coverslips.

For immunolabeling of synaptic density, array ribbons were immunostained with primary antibodies against total presynapses (synaptophysin) and presynapses from neurons overexpressing human tau (GFP) and fluorescently labeled secondary antibodies (Table 1). For immunolabeling of pathological tau spread, array ribbons were immunostained with primary antibodies against postsynapses (PSD95) and human tau (Tau13) and fluorescently labeled secondary antibodies (Table 1). Sections were counterstained with .01 mg/ml 4′‐6‐diamidino‐2‐phenylindole (DAPI). In each experiment, a short extra ribbon was used as a no primary negative control. For each area of interest (middle molecular layer of the dentate gyrus), images were obtained on serial sections using a Zeiss axio Imager Z2 epifluorescent microscope at 63X 1.4 NA Plan Apochromat objective with equipped CoolSnap digital camera and AxioImager software with array tomography macros (Carl Zeiss, Ltd, Cambridge, UK).

**Table 1:** Summary of antibody details used for array tomography

Primary antibody	Source	Dilution	Working concentration (mg/ml)	Secondary antibody
Synaptophysin	Abcam (ab8049)	1:50	.02	Donkey anti‐mouse Alexa594, Invitrogen
GFP	Abcam (ab13970)	1:100	.10	Goat anti‐chicken Alexa488, Abcam
Tau13	Covance (MMS‐520R)	1:50	.02	Donkey anti‐mouse Alexa647, Invitrogen
PSD95	Fronteir Institute (Af628)	1:10	.02	Donkey anti‐rabbit Alexa594, Invitrogen

Images from each set of serial sections were converted into image stacks and aligned using the Image J plug‐in, MultiStackReg (courtesy of Brad Busse and P. Thevenaz, Stanford University) (Thevenaz, Ruttimann, & Unser, 1998). Cropped regions of interest (10 μm^2^) within the middle molecular layer of the dentate gyrus were generated. Image stacks were then binarized using thresholding algorithms in ImageJ. For total presynaptic and postsynaptic images stacks were binarized using an ImageJ script that combines different thresholding algorithms to select both high and low intensity synapses in an automated and unbiased manner (macros are provided along with primary data contributing to the paper on the University of Edinburgh DataShare repository https://doi.org/10.7488/ds/1706). To calculate the synaptic density of both total presynapses and GFP‐positive presynapses, thresholded images were processed and analysed in MATLAB to remove background noise (objects present in only a single section were removed). The % GFP‐positive presynapses from each mouse was calculated as the sum of the number of GFP‐positive presynapses/sum total presynapses. To examine the spread of tau pathology, thresholded images were processed and analysed in MATLAB to remove background noise and to calculate the colocalization of Tau13 with postsynapses (a minimum of 50% overlap between Tau13 and PSD95 puncta was required to classify colocalization).

### 2.3 Statistical analyses

#### 2.3.1 Array tomography—synaptic density

Total presynaptic density detected by synaptophysin labeling and GFP‐positive presynaptic density were normally distributed across crops for each mouse, therefore, the mean total presynapses and mean GFP‐positive presynapses were taken for individual mice. Group total presynaptic density and GFP‐positive presynaptic density were normally distributed so a two‐way ANOVA was performed (SPSS). All values are reported as mean and *SEM*.

#### 2.3.2 Array tomography—spread of tau pathology

The % postsynapses colocalising with Tau13 from each mouse was calculated as the sum of the number of colocalization/sum total postsynapses within the MML. Group data for the colocalization of postsynapses with Tau13 was not normally distributed so the nonparametric Kruskal–Wallis test was performed (GraphPad Prism 5). The % synaptic pairs colocalising with Tau13 from each mouse was calculated as the number of pairs colocalising with Tau13/sum total synaptic pairs. Group data for the colocalization of synaptic pairs with Tau13 was not normally distributed so the nonparametric Kruskal–Wallis test was performed (GraphPad Prism 5). All values are reports as median and interquartile range.

## 3 Results

### 3.1 GFP‐positive synapse density is not altered prior to global synapse loss

**Figure 1:**
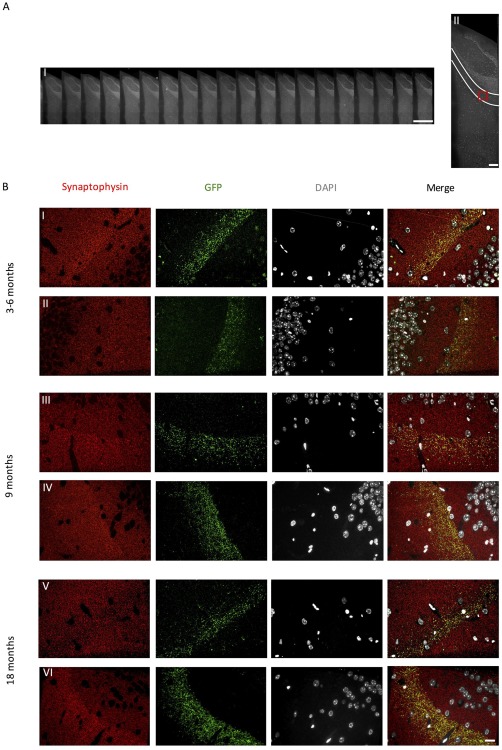
Synaptic images were obtained from the MML of the dentate gyrus from each section along the entire array ribbon (A.I–II). GFP expression is evident in the middle molecular layer of the dentate gyrus (B) of (I) 3–6 month (III) 9 month and (V) 18 month old rTgTauEC + EC‐tdTomato/Syp‐GFP mice and (II) 3–6 month (IV) 9 month and (VI) 18 month old EC‐tdTomato/Syp‐GFP control mice. Scale bars represent 1 mm in A(I), 200 μm in A(II), and 20 μm in B(I–VI)

Evident global synapse loss has previously been observed at 24 months of age in rTgTauEC mice (de Calignon et al., 2012). Synapse loss is preceded by behavioral abnormalities and presynaptic dysfunction at 16 months of age (Polydoro et al., 2014). Here, applying the high resolution imaging technique, array tomography, we examined whether synapse loss of the terminals that express mutant tau would precede the global synapse loss in the MML of the dentate gyrus in line with synaptic dysfunction. To characterize synaptic density a 1‐day imaging protocol was performed. To determine whether synaptic density of mutant tau terminals was altered with age we applied combinations of synaptophysin (total presynaptic recognising antibody) and GFP antibody (specifically recognising terminals from neurons overexpressing mutant tau and tdTom/EGFP (rTgTauEC + EC‐tdTomato/Syp‐GFP) or tdTom/EGFP alone (EC‐tdTomato/Syp‐GFP control). We observed strong GFP expression within the MML of 3–6, 9, and 18 month old mice of both genotypes (Figure 1).

**Figure 2:**
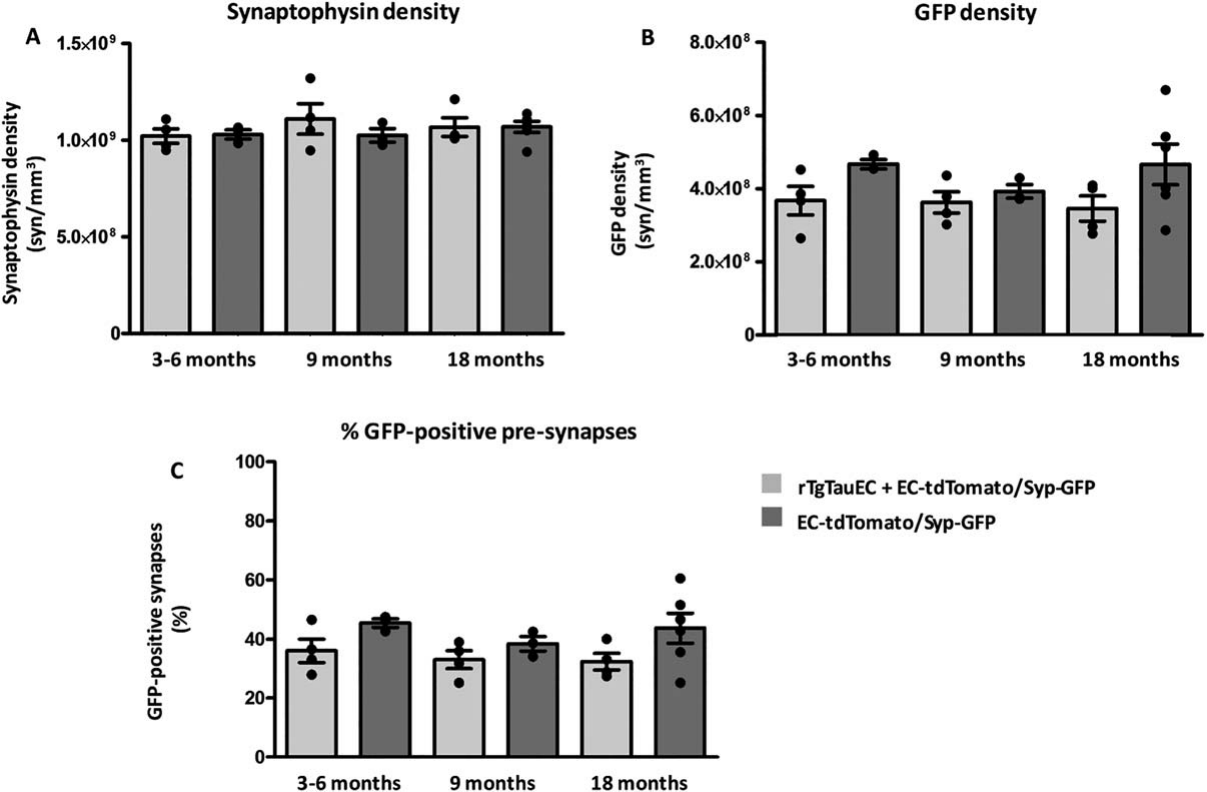
Quantification of synaptic density with array tomography. (A) total presynapses detected by synaptophysin immunolabeling, (B) GFP‐positive presynapses, (C) % GFP‐positive presynapses in the MML of the dentate gyrus. Data shown are means ± *SEM*, individual points represent the mean value from each mouse

Quantification of synaptic density revealed no significant effect of genotype or age on the density of global presynapses detected with synaptophysin labeling (Figure 2A). Examination of presynaptic terminals derived from neurons overexpressing mutant human tau revealed a significant effect of genotype (*p* < .05) on the density of GFP‐positive terminals (Figure 2B) and the percentage of total presynapses that were GFP‐positive (Figure 2C), however, there was no significant effect of age.

### 3.2 Array tomography reveals spread of tau prior to global synapse loss

**Figure 3:**
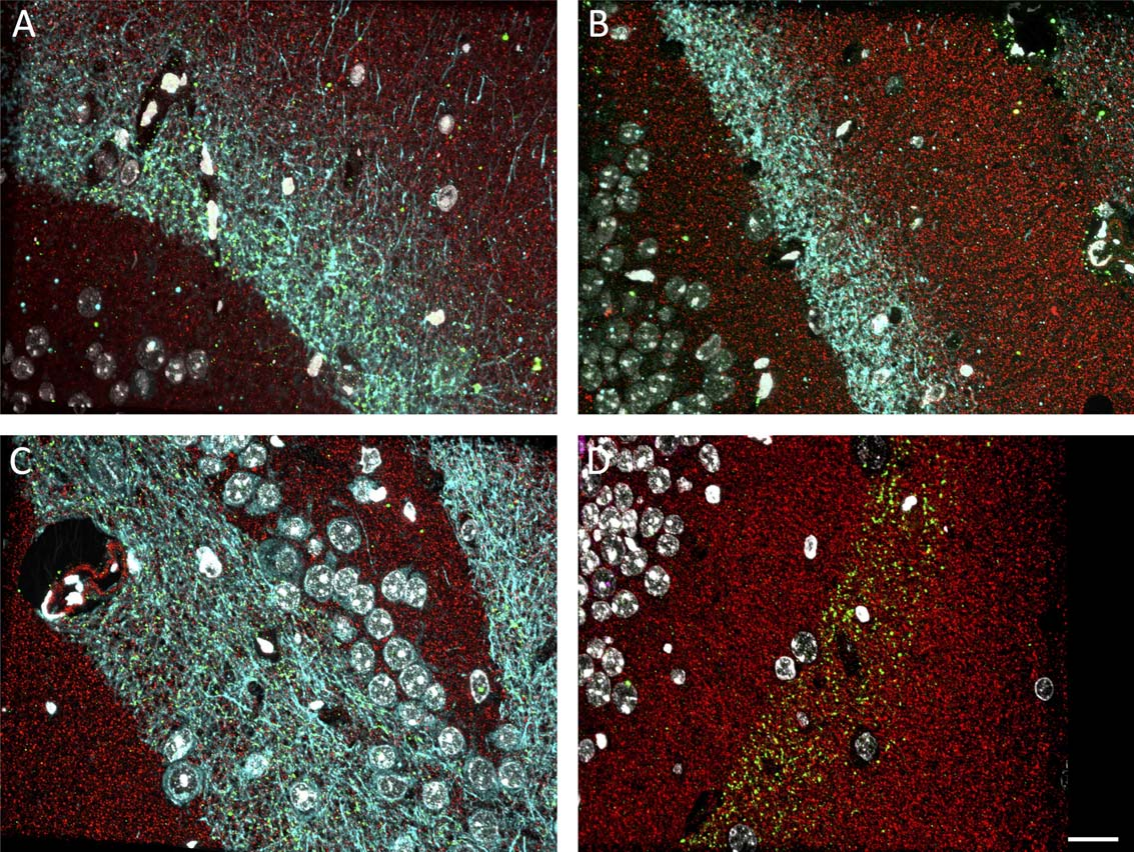
Human tau detected by Tau13 (cyan) in the MML of the dentate gyrus, Synaptophysin (red) and GFP (green) in (A) 3–6 month (B) 9 month (C) 18 month old rTgTauEC + EC‐tdTomato/Syp‐GFP mice. Human tau is not present in the MML of EC‐tdTomato/Syp‐GFP control mice (D). Scale bar represents 20 μm

Previous studies in rTgTauEC mice have shown granule cells of the dentate gyrus accumulating human tau at 18 months of age (de Calignon et al., 2012) while neuronal loss in the entorhinal cortex and global synapse loss are observed by 24 months of age. Here, we utilized a 1‐day imaging protocol to examine at what stage of disease progression tau protein spreads to the postsynapses of the MML. We observed human tau detected with Tau13 antibody within axons in the MML of the dentate gyrus of 3–6, 9, and 18 month old rTgTauEC + EC‐tdTomato/Syp‐GFP mice as expected (Figure 3A–C). Tau13 is not detected in red–green‐ EC‐tdTomato/Syp‐GFP control mice in which mutant tau is not overexpressed (Figure 3D).

**Figure 4:**
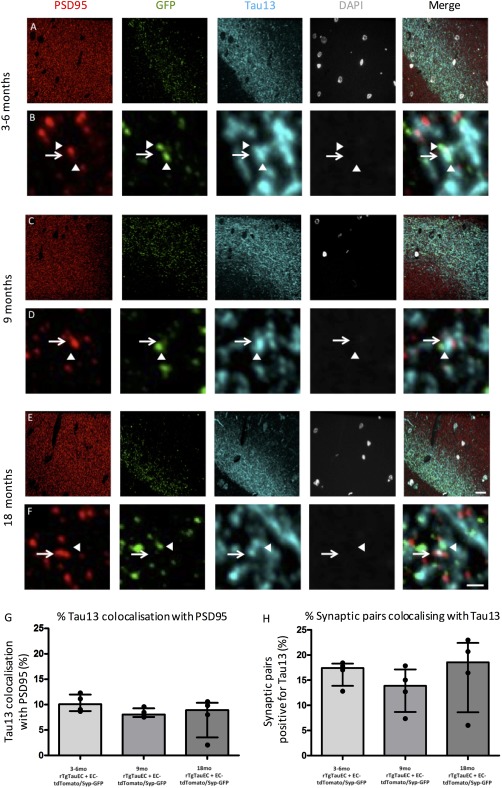
Human tau pathology spreads from presynaptic terminals derived from neurons overexpressing tau to the postsynapses of the MML of the dentate gyrus. Human tau pathology is present within the MML of 3–6 month (A) 9 month (C) and 18 month old (E) rTgTauEC + EC‐tdTomato/Syp‐GFP mice. Individual postsynapses detected by PSD95 are Tau13 positive at 3–6 months (B) 9 months (D) and 18 months (F). Arrows depict postsynapses, arrowheads depict neighboring presynapses derived from neurons overexpressing tau. Array tomography quantification reveals a subset of postsynapses (G) and a subset of putative synaptic pairs (H) colocalize with Tau13 at 3–6, 9, and 18 months of age. Scale bars represent 20 μm in a, c, e, and 1 μm in B,D,F. Data shown are medians and interquartile ranges; individual points represent the median value from each mouse

Further examination of postsynaptic puncta (PSD95 labeling) within the MML revealed the presence of human mutant tau at the postsynaptic density (Figure 4) of rTgTauEC + EC‐tdTomato/Syp‐GFP mice at all ages tested, starting at 3 months, which is 21 months prior to the loss of synapses that occur in this line (Figure 4A,B). Quantification within the middle molecular layer of the dentate gyrus revealed 10% of postsynapses at 3–6 months of age, 8% of postsynapse at 9 months of age and 9% of postsynapses at 18 months of age colocalising with Tau13 (Figure 4G). Further analysis of putative synaptic pairs (composed of presynapses derived from layer II EC neurons (GFP + ve), and postsynapses of the MML within 0.5 μm proximity), revealed a subset of synaptic partners in which both the presynaptic and postsynaptic compartments were tau positive at all ages studied (Figure 4H). This suggests that tau protein can spread from intact presynapses to neighboring postsynapses within this model without requiring presynaptic terminal degeneration.

## 4 Discussion

The spread of tau inclusions through the brain occurs in a well‐characterized hierarchal pattern in AD and plays a role in the disease pathogenesis. Despite accumulating evidence supporting trans‐synaptic propagation of tau through neuronal connectivity (de Calignon et al., 2012; Harris et al., 2012; Liu et al., 2012), the mechanism underpinning this pathological progression is not fully understood and remains a matter of debate. It has been proposed that degeneration of presynaptic terminals may result in the leakage of tau and subsequent spread to neighboring postsynapses (Wang & Mandelkow, 2016). Our data suggest that in the rTgTauEC + EC‐tdTomato/Syp‐GFP mouse model, the propagation of tau is not solely a consequence of axon terminal degeneration since tau is detected at postsynapses in the MML of the dentate gyrus prior to the loss of presynapses and in postsynapses directly opposed to presynaptic terminals. Our results are consistent with data from cultured primary rodent neurons and human iPSC derived neurons showing that tau is released from healthy neurons in the absence of cell death (Kanmert et al., 2015); and expand this concept demonstrating that *in vivo*, tau can spread from presynaptic to postsynaptic elements without substantial loss of presynaptic terminals.

Several key questions remain to understand tau propagation through the brain including, which types of tau spread and what are the mechanisms of tau release from presynapses and uptake from postsynapses? Many forms of tau have been observed to be secreted in vitro including phosphorylated tau (Pooler, Phillips, et al., 2013; Saman et al., 2012) and C‐terminally truncated tau (Kanmert et al., 2015). Recent data from AD cerebrospinal fluid and brain samples indicates that high molecular weight tau species may be released from human neurons and are competent to induce tau seeding in cultured cells (Takeda et al., 2016). Several mechanisms of tau release have been proposed (reviewed by (Wang & Mandelkow, 2016)) including exocytosis of tau, vesicle mediated release such as in exosomes (Saman et al., 2012), or synaptic vesicle release (Polanco, Scicluna, Hill, & Gotz, 2016). Once released from the presynaptic cell, multiple mechanisms could also regulate uptake in recipient neurons including endocytosis or fusing of exosomes with recipient cells. In addition to neuronal release and uptake of tau, there is evidence for a role of glia in modulating tau spreading. Microglial activation has been reported to precede tau propagation (Maphis et al., 2015) and it has been proposed that microglia act to phagocytise and release tau in microglial‐derived exosomes (Asai et al., 2015).

The uptake of tau by neighboring postsynapses ultimately induces neurofibrillary tangles within downstream neurons via as yet unknown templating or seeding mechanisms. These intracellular inclusions correlate with the cognitive decline and neuronal loss observed in AD (Arriagada, Growdon, Hedley‐Whyte, & Hyman, 1992; Giannakopoulos et al., 2003). However, neuronal loss greatly exceeds the burden of neurofibrillary tangles, and it has become increasingly recognized that soluble forms of tau may mediate synaptic and neuronal toxicity (Gomez‐Isla et al., 1997; Kopeikina, Hyman, & Spires‐Jones, 2012; Lasagna‐Reeves et al., 2011; Rocher et al., 2010).

Despite the many questions that remain surrounding the propagation of tau through the brain, it is clear that tau spreads through the brain, likely via synaptic circuits. A promising therapeutic avenue is to prevent the spread of tau using immunotherapies (Gerson & Kayed, 2016). Our results indicate that this spread begins very early in the disease process, and shows the utility of the array tomography technique for detecting tau protein at the level of individual synapses. This will be useful in preclinical studies of treatments to determine whether they prevent the trans‐synaptic spread of tau.
